# Changes in Anti–OV-16 IgG4 Responses to Onchocerciasis after Elimination of Transmission in the Central Endemic Zone of Guatemala

**DOI:** 10.4269/ajtmh.23-0473

**Published:** 2024-03-19

**Authors:** Vitaliano A. Cama, Renata Mendizabal-Cabrera, Oscar de Leon, Michael White, Circe McDonald, Elizabeth Thiele, Guilherme M. Ogawa, Zoraida Morales, Jessica Prince-Guerra, Paul Cantey, Nidia Rizzo

**Affiliations:** ^1^ Global Health Center, Centers for Disease Control and Prevention, Atlanta, Georgia;; ^2^ Centro de Estudios en Salud, Universidad del Valle de Guatemala, Guatemala City, Guatemala;; ^3^ Infectious Disease Epidemiology and Analytics G5 Unit, Institut Pasteur, Université Paris Cité, Paris, France;; ^4^ Onchocerciasis Sub-Program, Ministerio de Salud Publica y Asistencia Social, Ciudad de Guatemala, Guatemala

## Abstract

Current WHO guidelines for onchocerciasis elimination provide requirements for stopping mass drug administration of ivermectin and the verification of elimination of transmission. These guidelines also recommend post-elimination surveillance (PES) based on entomological surveys. Serological markers in humans could complement entomological PES once the longevity of anti–OV-16 antibody responses is better understood. In 2014–2015 we evaluated ELISA anti–OV-16 IgG4 antibody persistence among previously seropositive people from the central endemic zone of Guatemala. The country stopped all onchocerciasis program interventions in 2012 and was verified by WHO as having eliminated transmission of onchocerciasis in 2016. A total of 246 participants with prior OV-16 ELISA results from 2003, 2006, 2007, or 2009 were enrolled in a follow-up study. Of these, 77 people were previously OV-16 seropositive and 169 were previously seronegative. By 2014 and 2015, 56 (72.7%) previously seropositive individuals had sero-reverted, whereas all previous negatives remained seronegative. The progression of antibody responses over time was estimated using a mixed-effects linear regression model, using data from seropositive participants who had sero-reverted. The temporal variation showed a mean activity unit decay of 0.20 per year (95% credible interval [CrI]: 0.17, 0.23), corresponding to an estimated antibody response half-life of 3.3 years (95% CrI: 2.7, 4.1). These findings indicate that the majority of seropositive people will sero-revert over time.

## INTRODUCTION

Onchocerciasis, or river blindness, is a vector-borne neglected tropical disease caused by the nematode *Onchocerca volvulus*. Currently, it is estimated that 20.9 million people are infected, and approximately 205 million are at risk of acquiring the disease,^1^ mainly in sub-Saharan Africa and Yemen. Onchocerciasis is targeted for elimination primarily through mass drug administration (MDA) of Mectizan. In the Americas, the transmission of onchocerciasis has been eliminated in 11 of the 13 historically endemic foci, and four countries have been verified by WHO as having interrupted the transmission of onchocerciasis: Colombia in 2013,^2^ Ecuador in 2014,^3^ México in 2015,^4^ and Guatemala in 2016.^5^

According to the current WHO guidelines for onchocerciasis elimination, national programs should develop a post-elimination surveillance (PES) method for monitoring for recrudescence or reintroduction of the disease.^6^ The guidelines suggest the implementation of entomological surveys at regular intervals in formerly endemic and nonendemic regions. Guidance for the recommended number and location of sites for simulid collection, number of flies to collect, and frequency of fly collection has not been developed.

Interruption of transmission requires that the incidence of new patent adult worm infections be reduced below the theoretical breakpoint for maintaining the life cycle, R_0_. Because there are uncertainties about both the actual breakpoint for any particular location and the prevalence of infection in humans and flies when MDA is stopped, studies assessing the risk of recrudescence as well as the risk of reintroduction of infection are needed to inform strategies for surveillance.

Better understanding of the temporal evolution of the human antibody response to *O. volvulus* is also needed to inform serological strategies for PES. Studies in nonhuman primates have shown that IgG4 antibody responses to parasite antigen OV-16 were detectable at 15 months after inoculation. And after more than 4 years of follow-up, there was a trend for antibody clearance (sero-reversion).^7^ Data from a 10-year follow-up study of onchocerciasis patients residing in the United States also showed sero-reversion of anti–OV-16 responses in half the patients.^8^ More recently, a study in Nigeria comparing onchocerciasis microfiladermia in communities that received 17 years of annual ivermectin MDA demonstrated declines in OV-16 seropositivity, suggesting that sero-reversions had likely occurred.^9^

In this study, we investigated changes in anti–OV-16 IgG4 antibody reactivity among individual residents of formerly endemic areas in central Guatemala (the “central endemic zone”) before and after onchocerciasis transmission was eliminated to provide the first specific estimates of longevity of antibody responses and rates of antibody decay.

## MATERIALS AND METHODS

### Study site.

The study was conducted in four communities from the departments of Suchitepéquez and Chimaltenango in the onchocerciasis central endemic zone in southwestern Guatemala. The study included two yearly visits, the first one in 2014 and a follow-up visit in 2015. In this focus, annual ivermectin MDA started in the 1980s.^5^ The national onchocerciasis program implemented twice-yearly MDA with ivermectin in 2000 and reached more than 85% coverage of the eligible population in 2002. This strategy was maintained until MDA was suspended in 2012 after entomological and serological surveys found no infective blackflies and no children under 10 years old positive for IgG4 antibodies against OV-16. We selected this site because of the unique opportunity to study individual changes in antibody levels given that 1) the area used to be hyperendemic for onchocerciasis and transmission no longer existed in this focus and 2) because transmission had been interrupted, any person with previous seropositive results would not have been exposed to or reinfected with *O. volvulus* over the past 3 years.

### Study population.

Historical data from surveys conducted by the National Ministry of Health (NMH) identified 193 people of all ages with positive anti–OV-16 serology between 2003 and 2009. These program surveys were not part of the assessments to determine if transmission of onchocerciasis was interrupted. Our study aimed to enroll two seronegative persons for each seropositive individual. This was based on the assumption of 30% onchocerciasis exposure among seronegative individuals before interruption of transmission was verified and a significance level of 95%. This gave the study 90% power to detect new seroconversions among previously seronegative persons. For study inclusion, participants also had to have been permanent residents of their communities for ≥5 years.

### Questionnaire.

Basic demographic and epidemiological information about participants (age, sex, community, years of residence in the endemic area, history of treatment with ivermectin, self-reported adherence to treatment, and presence of skin or eye symptoms) was collected using questionnaires on smart phones.

#### Sample collection.

Peripheral venous blood was collected from study participants in heparinized Vacutainer^™^ tubes. Dried blood spots (DBSs) were prepared immediately after collection by spotting 70* µ*L of whole blood on Whatman 903 paper (GE Healthcare Bio-Sciences, Pittsburgh, PA). Participants unwilling to provide blood samples by venipuncture but willing to participate in the study had the option of providing a blood sample by finger prick.

#### OV-16 ELISA.

IgG4 antibody responses against *O. volvulus* OV-16 antigen were tested by ELISA following the same methodology previously used in Guatemala.^10^^,^^11^ Briefly, 96-well Immulon II plates (Thermo Fisher Scientific, Waltham, MA) were coated with OV-16 antigen at a concentration of 0.1* µ*g/well in 100* µ*L of carbonate buffer (NaHCO_3_ 0.1 M, pH 9.6) and incubated overnight at 4°C. Plates were washed 3× with phosphate buffered saline–Tween (PBST) (0.025 M, pH 7.0–7.4, 0.05% Tween 20.) Two 6-mm punches from each DBS were incubated overnight at 4°C in 200* µ*L of PBST containing 5% bovine serum albumin (BSA) (Sigma Chemical Co., St. Louis, MO). The next day, the plates were washed 4× with PBST, followed by blocking for 1 hour at 4°C with 100* µ*L of PBST-BSA. The blocking solution was decanted, the plates were washed 2× with PBST, and 50* µ*L of either assay controls or experimental samples was added to duplicate wells and incubated for 2 hours at room temperature (RT). Samples were decanted, and the plates were washed 4× with PBST. Fifty microliters per well of biotin-conjugated monoclonal mouse anti-human IgG4 (Invitrogen Corporation [previously Zymed Corp], Camarillo, CA) at a 1:1,000 dilution in PBST was added per well and incubated for 1 hour at RT. Plates were washed 4× with PBST and incubated for 1 hour at RT with 50* µ*L of streptavidin-alkaline phosphatase (Invitrogen) diluted 1:100 in PBST. Before substrate was added, plates were washed 4× with PBST. The ELISA was developed with 50* µ*L per well of p-nitro phenyl phosphate (Sigma) dissolved at 1 mg/mL in development buffer (10% diethylonamine, 3 mM MgCl, pH 9.8). The plates were incubated at RT and monitored for chromogen development until the wells with the 1:20 standard dilution reached an optical density (OD) value of 1.500. The reaction was stopped with sodium hydroxide 1 N, and the plate was read in an ELISA reader set at 405 nm.

### Standard curve and cutoff determination.

A reference standard for the quantification of the results was developed previously by the onchocerciasis laboratory group at Universidad del Valle de Guatemala (UVG) to normalize results and control for inter-assay variability. This protocol has been used in all serological evaluations in the region by the Onchocerciasis Elimination Program for the Americas since 2003. The standard curve was generated by serial dilutions of a pool of positive samples previously collected in Guatemala. The same protocol and normalizing positive control were used in our study. Briefly, each of the selected dilutions was assigned an activity unit (AU) value, starting with an arbitrary value of 2,500 units that was assigned to the 1:10 dilution. The resulting AUs were plotted against OD values and fit to a standard curve using the four-point parameter logistic function from SoftMax-Pro v. 4 (Molecular Devices, Sunnyvale, CA). The optimal threshold values for positivity (cutoffs) were identified using receiver operating characteristic (ROC) analyses. The ROC analyses used samples from people with confirmed infections (had at least one onchocercoma), and negatives were from people living in similar but nonendemic settings and from people with other parasitic diseases: lymphatic filariasis, schistosomiasis, and *Taenia solium* cysticercosis (Supplemental Tables 1 and 2). Using the previously determined 40 AU cutoff (Supplemental Figure 1), this OV-16 ELISA had a sensitivity of at least 60% and a specificity of at least 99% (Supplemental Table 1).

### IgG4 antibody decay model.

For these analyses, it was assumed that anti–OV-16 IgG4 antibody values would decay exponentially over time, which is equivalent to a linear reduction of antibody titer on a log scale. A mixed-effects model was fitted to the data from each person to account for individual variation in antibody decay rates. Only data from people with AU values that decreased from the endemic period (2003–2009) to the samples collected in this study (2014 or 2015) were used in this model.

The antibody level *A_ij_* in individual *i* at time point *t_j_* is given by

$$\text{log}\left(A_{ij}\right)∼\text{log}\left(A_{i,2006}\right)-\left(r+r_{i}\right)t_{j}+\varepsilon_{\text{ij}},$$

where *A_i_*_,2006_ is the antibody level in 2006, *r* is the average rate of decay of antibody level in the population, *r_i_* ∼ *N*(0, σ*_r_*) is the deviation in the decay rate of individual *i* from the population average, and ε_ij_ ∼ *N*(0, σ_obs_) is the normally distributed measurement error. The model was fitted to the data in a Bayesian framework using uniform priors defined as follows: *r* ∼ *U*(0, 100), σ*_r_* ∼ *U*(0, 100), and σ_obs_ ∼ *U*(0, 100).

#### Protection of human subjects.

The study protocol, consent and assent forms, and questionnaire received ethical approval from the institutional review board of the CDC (protocol no. 6565), the Ethics Committee of the Center for Health studies, UVG (protocol 092-01-2014), and the National Committee of Health Ethics of the NMH of Guatemala (protocol 39-2014).

## RESULTS

### Enrollment and demographics.

A total of 246 people were enrolled in the study, median age 34 years (range: 8–91 years). At time of enrollment, the median reported time living in the community was 27 years (range: 8–65 years). Of the 193 people who were seropositive in surveys conducted between 2003 and 2009 (Table 1), we were able to locate and enroll a total of 77 individuals, of whom 71 were enrolled in 2014 with six new enrollees in 2015. Among persons identified as seronegative in 2003–2009, we were able to enroll 169 persons, for a total recruitment of 246 people. The study participants were enrolled from the communities of Los Andes (*n =* 76, 31%), La Estrellita (*n =* 73, 30%), Montecarlo (*n =* 31, 13%), and Santa Isabel (*n =* 66, 27%). There were 124 females (50%) and 122 males (50%) (Table 1).

**Table 1:** Demographics and original OV-16 serological status of the 246 study participants

Characteristics of Study Participants	OV-16(+)	OV-16(−)	Totals
Year originally diagnosed, number (%)			
2014	71 (31%)	158 (69%)	229
2015	6 (35%)	11 (65%)	17
Total	77 (31%)	169 (69%)	246
Community, number			
Los Andes	14	62	76
La Estrellita	33	40	73
Montecarlo	11	20	31
Santa Isabel	19	47	66
Sex, number			
Female	38	86	124
Male	39	83	122
Age (years)			
Median	41	23	33
Range	12–76	8–91	8–91

Previously seropositive participants contributed 89 OV-16 ELISA data points from the endemic period (2003–2009) and 127 from the post-endemic period (2014–2015). Seronegative people contributed 221 OV-16 ELISA data points from 2003–2009 and 266 samples for the post-endemic period of 2014–2015 (Table 2).

**Table 2:** Number of samples with prior OV-16 ELISA results (2003–2009) and number of samples collected for this study (2014–2015)

Number of Samples from Study Participants	2003–2009	2014–2015
Seropositive (*n* = 77 people)		
One sample	65	27
Two samples	12	50
Total no. of samples	89	127
Seronegative (*n* = 169 people)		
One sample	115	72
Two samples	53	97
Total no. of samples	221	266

### Questionnaire at time of enrollment.

All 246 participants reported that ivermectin had been distributed in their communities prior to 2012 and that they were treated at least once in the past. The vast majority (95%) reported having been bitten by blackflies (“mosca canche”) in the past year. Responses to questions about clinical manifestations are presented in Table 3.

**Table 3:** Reported symptoms and exposure history from the epidemiological questionnaires (2014–2015)

Factors	Participant Responses	Seropositive	Seronegative	Total	%
IVM ever distributed	Yes	77	169	246	100
	No	–	–	–	–
Ever taken IVM	Yes	77	196	246	100
	No	–	–	–	–
Remembered last time IVM taken	Yes	8	15	23	9.3
	No	66	152	218	88.6
	Don’t know	3	2	5	2.0
Bitten by blackflies in past year	Yes	71	163	234	95.1
	No	6	6	12	4.9
Skin and visual manifestations					
Nodules (self-reporting)	Yes	7	4	11	4.5
	No	69	164	233	94.7
	No response	1	1	2	0.8
No. of nodules per person (*N* = 11 people)					
	One nodule	6	4	10	4.1
	Three nodules	1	0	1	0.4
Skin changes noted	Yes	4	7	11	4.5
	No	72	160	232	94.2
	Don’t know	1	2	3	1.2
Types of skin changes					
	Depigmentation	1	1	2	0.8
	Other	3	6	9	3.6
Itching frequency	Daily	9	9	18	7.3
	Weekly	1	1	2	0.8
	Monthly	6	4	10	4.1
	Less than once a month	55	145	200	81.3
	Don’t know	6	10	16	6.5
Changes in vision	Yes	30	35	65	26.4
	No	46	134	180	73.2
	Don’t know	1	0	1	0.4
Specific changes in vision					
	Blurry	18	17	35	14.2
	Nearsightedness/farsightedness	7	7	14	5.7
	Tears, itch, burn, pain	7	5	12	4.9
	Other	2	2	4	1.6

IVM = ivermectin.

#### OV-16 ELISA reactivity.

**Figure 1.**
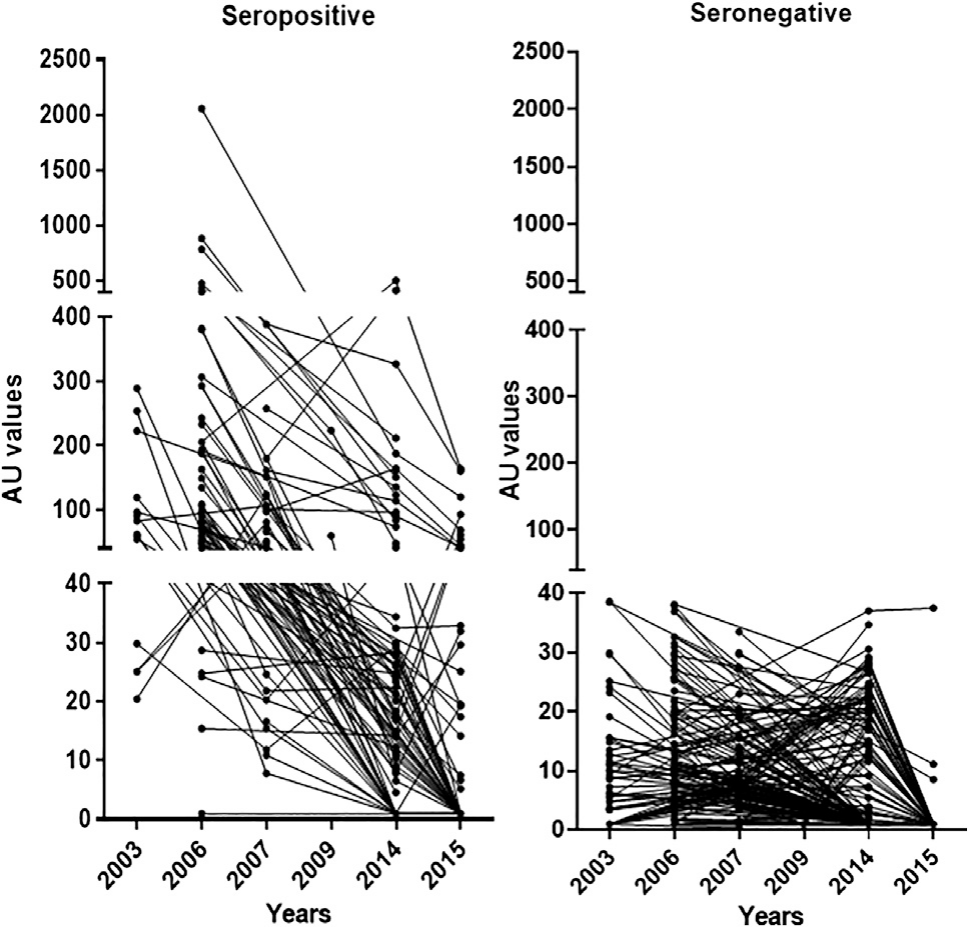
Individual OV-16 ELISA AU from study participants at different years of serological surveillance: samples from 2003–2009 when onchocerciasis was endemic; samples from 2014 and 2015 after interruption of transmission, with 40 AU as the threshold for OV-16 ELISA seropositivity. AU = activity unit.

In the seropositive group, the OV-16 ELISA values during 2003*–*2009 had median AU ranging between 83 and 141 AU, whereas the range of medians among seronegative participants was 1.0–8.7 AU (Table 4). During 2014*–*2015, 56 initially seropositive individuals (72.7%) had reverted to negative OV-16 ELISA results (AU <40). All participants who were seronegative in 2003*–*2009 remained antibody negative (Table 4). Overall, data from seropositive participants showed a declining trend in AU over time (Figure 1).

**Table 4:** OV-16 ELISA AU among seropositive and seronegative groups by year of testing

OV-16 ELISA Responses Among Study Participants	Results by Year from Previous Program Assessments (endemic onchocerciasis transmission ongoing)				Results from this Study (post-endemic)	
	2003	2006	2007	2009	2014	2015
Seropositive (number)	13	51	23	2	71	56
AU median	83.0	97.5	70.7	141.6	18.4	1.0
AU range	20.4–268.7	40.7–2,055	7.8–388.9	59.5–223.7	1–503.4	1–164.1
AU mean	107.7	217.3	96.7	141.6	51.6	19.5
Seronegative (number)	50	78	78	15	158	108
AU median	8.7	12.5	7.8	1.0	1.0	1.0
AU range	1–38.6	1–38.1	1–33.5	1	1–37.0	1–37.5
AU mean	10.6	15.3	10.0	1.0	9.4	1.7
OV-16 sero-reversion					2014 (*N* = 71)	2015 (*N* = 56)
Number of new sero-reversions by year of detection					54	2
Total (%)					56 (72.7%)	

AU = activity units.

### Evolution of antibody responses over time.

#### Rate of antibody decay in previously seropositive individuals.

**Figure 2.**
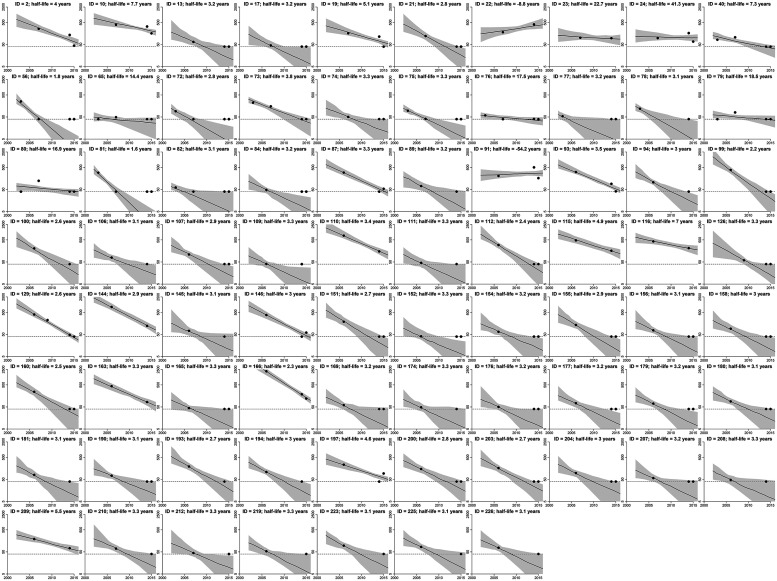
Individual level anti–OV-16 antibody dynamics for 77 individuals with at least two measurements of antibody response (red points) as used for modeling. The dashed line shows the cutoff of 40 AU below which antibody responses were not censored, and those values were recoded as 40 AU. The black lines show the model estimated antibody titer. The grey region denotes the 95% credible interval. Note that because antibody responses cannot be reliably measured below the threshold of 40 AU, any measurement with a value <40 AU is plotted on the dashed line. AU = activity unit.

**Figure 3.**
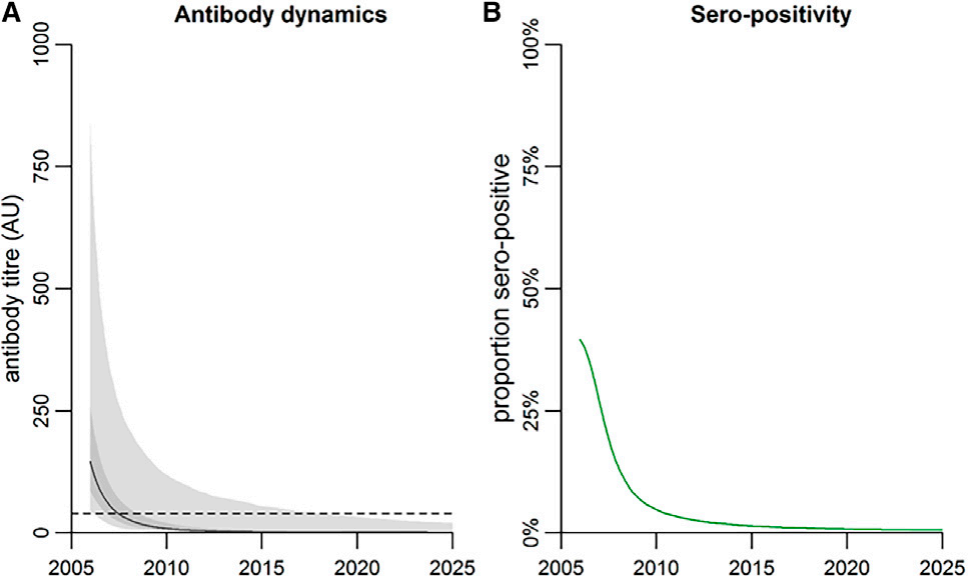
Population-level anti–OV-16 antibody dynamics. (**A**) Predicted change in median anti–OV-16 antibody response over time in participants who were seropositive in 2006. The dark and light grey shaded areas show the 50% and 95% ranges of the data. (**B**) Predicted change in anti–OV-16 seropositivity over time was defined as the proportion of the population with antibody titers >40 AU. AU = activity units. Dashed line represents 40 AU, the cutoff for OV-16 ELISA sero-positivity.

There was variation among individual decay patterns (Figure 2). Using data from the 56 people with sero-reversion, the estimated mean rate of decay of antibody levels was estimated at 0.198 AU (95% credible interval [CrI]: 0.166, 0.233) per year. The SD in decay rates between the 56 seropositive individuals was estimated at 0.115 (95% CrI: 0.083, 0.153) per year. This is equal to an average half-life of antibody decay in the sero-reverted population of 3.3 (95% CrI: 2.7, 4.1) years. Figure 3A shows the estimated antibody dynamics of AU for the 56 people who sero-reverted with simulation until 2025.

#### Seroprevalence rates in the community.

The rate of antibody decay was applied to determine its effect on seroprevalence in the communities in this study. Figure 3B shows the estimated seropositivity rates from 2006 until 2025. In 2006, 39.5% of individuals were seropositive. Based on the AU levels detected in this study population, we estimated that half of these seropositive individuals sero-reverted after 1.4 years. Therefore, we estimate the community sero-reversion half-life for this study to be 1.4 years.

Of the 77 seropositive individuals in the study, 55 were diagnosed by ELISA only, whereas 22 of ELISA-positive individuals were also positive for microfilariae (MF) in skin snips. From these previous results, the 22 participants with MF-positive results had significantly higher antibody titers (median *=* 345 AU, mean *=* 508 AU) compared with those who had only serology-positive results (median *=* 147 AU, mean = 295 AU) (*P =* 0.0013, two-sided *t*-test). However, when we analyzed the waning of antibody titers over time, there were no statistically significant differences in the rates of decay between individuals with positive ELISA and MF (median *=* 0.23, mean = 0.22) and those positive only by serology (median *=* 0.22, mean = 0.20) (*P =* 0.40, two-sided *t*-test) (Supplemental Figure 2). These findings suggest that individuals who were MF positive had stronger antibody responses; however, it did not affect the rates of antibody decay.

## DISCUSSION

There are few studies on the longitudinal dynamics of antibody responses against onchocerciasis. Most studies focused on the time to seroconversion, with reports of first anti–OV-16 IgG seropositivity at 13 months^12^ and IgG4 at 15 months^7^ after inoculation of nonhuman primates in laboratory-controlled environments. However, understanding the dynamics of antibody decay is very important as well. Current WHO guidelines for the verification of the elimination of transmission of onchocerciasis require long-term surveillance until regional elimination occurs. Although guidelines currently recommend only the use of entomological assessments, it may be difficult to maintain entomological surveillance after the initial posttreatment surveillance period is completed. We believe that monitoring antibody levels may be a more feasible way to perform PES in the future.

Measurement of OV-16 IgG4 has been used to demonstrate the interruption of transmission of onchocerciasis in both the Americas^2^^,^^10^^,^^13^^–^^19^ and Africa.^20^ However, it cannot distinguish between resolved infections that no longer contribute to transmission and new or current infections that may contribute to transmission. Understanding the rates of antibody decay for onchocerciasis may allow the use of human serology as a surveillance tool. Serologic surveillance could potentially be conducted across an expanded range of age groups, which would overcome a limitation of the current monitoring strategy, which focuses on serologic testing of people born after onchocerciasis elimination programs were implemented.

The use of serology may offer some additional advantages, as it may be easier to conduct assessments on human populations than to conduct an onchocerciasis-specific entomological assessment. In general, health workers are more familiar with blood sample collection than with identification of breeding sites and capture of *Simulium* vectors. Another potential advantage is that sero-surveys for onchocerciasis could be integrated with other public health surveillance activities, either through collection of specimens that could be tested by national onchocerciasis programs or as part of integrated sero-surveillance activities in which testing could be done using multiplex bead assays, which can simultaneously measure antibody responses to onchocerciasis and other pathogens.^21^ Overall, serology could be a more feasible and informative option for onchocerciasis surveillance in the post-elimination period.

Our findings have some potential limitations. Identifying and enrolling people from surveys conducted 5 or more years ago was very challenging, and the number of study participants recruited (*N =* 246) was relatively small. However, this sample size is substantially larger than those of previous studies of antibody dynamics^8^ and was large enough that we were able to estimate rates of decay. It also would have been ideal to have all samples tested simultaneously. However, the results from the sero-surveys previously conducted by the NMH were tested in the same laboratory at UVG, following the same protocol. Furthermore, all OV-16 ELISA AU values were normalized with the same reference controls as used between 2003 and 2009. Therefore, the OV-16 ELISA AU values determined in 2014 and 2015 were likely comparable to AU values determined in 2003–2009, allowing us to model the dynamics of sero-reversion.

We found that a substantial number of formerly seropositive study participants sero-reverted by 2014. This study also determined that the rates of decay were similar regardless of a positive diagnosis for MF, suggesting that in this study population, detection of parasites at baseline would lead to higher ELISA antibody titers, although it did not appear to affect the overall rates of antibody waning.

The seropositive participants, whose data were used to determine the rates of decay, were mostly adults whose ages were greater than those recommended in the WHO guidelines for elimination of onchocerciasis.^6^ However, more than 70% had sero-reverted. This observation may lead to future studies to determine expanded age ranges for PES activities that could provide the necessary evidence for revised PES guidelines. Despite sampling an older age population, our findings show a strong trend to sero-reversion in this community.

As significant progress is made toward the elimination of onchocerciasis, with >90% reduction in the population at risk in the Americas and multiple foci showing interruption of transmission in African settings, there is a clear need to define strategies for PES that could complement or substitute for the vector monitoring activities proposed in the current WHO guidelines. Serology for PES could indeed be more feasible to implement, and further studies will be needed to provide evidence for making future recommendations. For example, studies that generate community representative disease exposure over time, such as age-seroprevalence curves, could provide key information to determine the dynamics of seroconversion by age and reliable data for new age ranges for serology-based PES. These studies not only would broaden the age range for individuals to be surveyed but could also increase the quality, feasibility, and reliability of PES.

Our data on the dynamics of sero-reversion suggest that serology assays such as OV-16 ELISA can be reliably used for programmatic assessments and can provide preliminary evidence for using serological assessments after the successful elimination of onchocerciasis transmission.

## Supplemental Materials

10.4269/ajtmh.23-0473 Supplemental Materials
