# Supplementary material for: Changes in Anti–OV-16 IgG4 Responses to Onchocerciasis after Elimination of Transmission in the Central Endemic Zone of Guatemala
Source: Am J Trop Med Hyg. 2024 Mar 19;110(5):943–50. doi: 10.4269/ajtmh.23-0473 (PMC11066353; doi:10.4269/ajtmh.23-0473)

Supplemental Table 1: Characteristics of the specimens used for assay development (2004). Dry blood spots abbreviated as DBS

Type of sample	Criteria	Number of samples		OV-16 ELISA positive results
A. Assay development, determination of standard curve and cut off value				
Positive	Presence of ≥ 1 nodule	Sera	62	38 (61%)
		DBS	25	15 (60%)
		Total	87	53 (60.9%)
Negative	Non endemic area	Sera	19	0
		DBS	355	1 (0.3%)
		Total	374	1(0.3%)
B. OV-16 ELISA versus skin snip microscopy				
Positive	Presence of microfilariae [MF] (skin snips from scapula or iliac crest)	33		28 (84.8%)
Negative	MF negative	29		12 (41.4%)
	Total:	62		
C. Performance against a blind panel of samples				
Positive	MF+ or nodule positive	18		11 (61.1%)
Negative	Schistosomiasis	6		0
	Lymphatic filariasis	6		0
	T. solium cysticercosis	5		0
	Non endemic USA	7		0

	Total	42	
D. Post-Development Performance on a community survey			
Site 1	Meso endemic	85	26 (30.6%)
Site 2	Meso endemic	87	45 (52.9%)
Site 3	Meso endemic	70	16 (22.9%)
Site 4	Meso endemic	86	26 (30.2%)
	Total endemic	328	113 (34.4%)
Site 5	Non endemic	93	0
Site 6	Non endemic	73	0
Site 7	Non endemic	83	0
Site 8	Non endemic	99	1 (1%)
	Total non-endemic	348	1 (0.3%)

Supplemental Table 2: Specimens used for the determination of cut-offs by Receiver Operator Characteristics (ROC) analysis.

Initial development and determination of standard curve				
Positive	Presence of ≥ 1 nodule	Sera	62	38 (61%)
		DBS	25	15 (60%)
		total	87	53 (60.9%)
Negative	Non endemic area	Sera	19	0
		DBS	355	1 (0.3%)
		Total	374	1(0.3%)

Supplemental Table 3. Number of people with nodules or microfilariae detected between 2003 and 2009 and enrolled in 2014 and 2015. The columns describe total number people surveyed in each year; number enrolled for this report including serological classification. Numbers in parentheses are the percentages relative to the total number examined by column.

	2003				2006				2007				2009			
Group	Survey	This study	sero-positive	sero-negative	Survey	This tudy	Sero-positive	Sero-negative	Survey	This study	Sero-positive	Sero-negative	Survey	This study	Sero-positive	Sero-negative
Presence nodules	31 (25)	11 (18)	4 (31)	7 (15)	32 (25)	15 (14)	14 (28)	1 (3)	13 (13)	13 (13)	6 (30)	7 (9)	8 (15)	3 (21)	1 (50)	2 (17)
Microfilariae (MF) positive	7 (6)	1 (2)	0	1 (2)	7 (6)	7 (7)	6 (12)	1 (2)	0	0	0	0	1 (2)	0	0	0
Nodule MF (+)	11 (9)	0	0	0	7 (6)	5 (5)	5 (10)	0	0	0	0	0	1 (2)	0	0	0
Negative	77 (61)	48 (80)	9 (69)	39 (83)	81 (64)	79 (75)	25 (50)	54 (96)	85 (87)	84 (87)	14 (70)	70 (91)	43 (81)	11 (79)	1 (50)	10 (83)
Total examined	126	60	13	47	127	106	50	56	98	97	20	77	53	14	2	12

Supplemental Figure 1: ROC determination of the cutoff at 40 activity units (OD about 0.05).

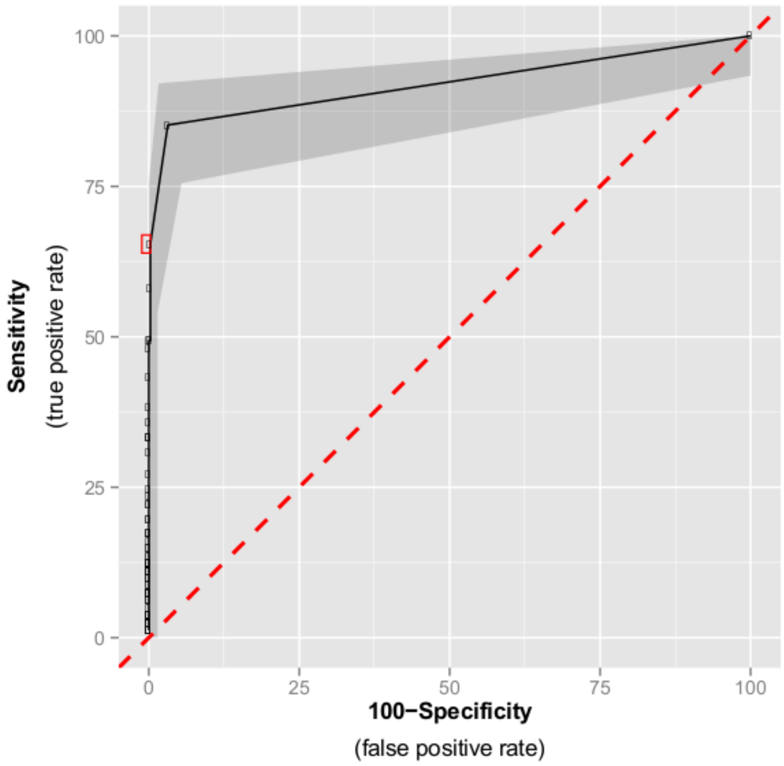

Supplemental Figure 2: Boxplots comparing antibody waning and initial antibody titers among OV-16 serology positive individuals. Group “OV-16” represents individuals positive to serology alone, and “OV MF” represents those positive by serology and microfilariae in skin snips. Pane 2a shows the estimated rates of waning of anti-OV-16 antibody responses (no statistically significant differences, p = 0.40). Pane 2b shows antibody titers by group (statistically significant difference, p=0.0013).2a

2b

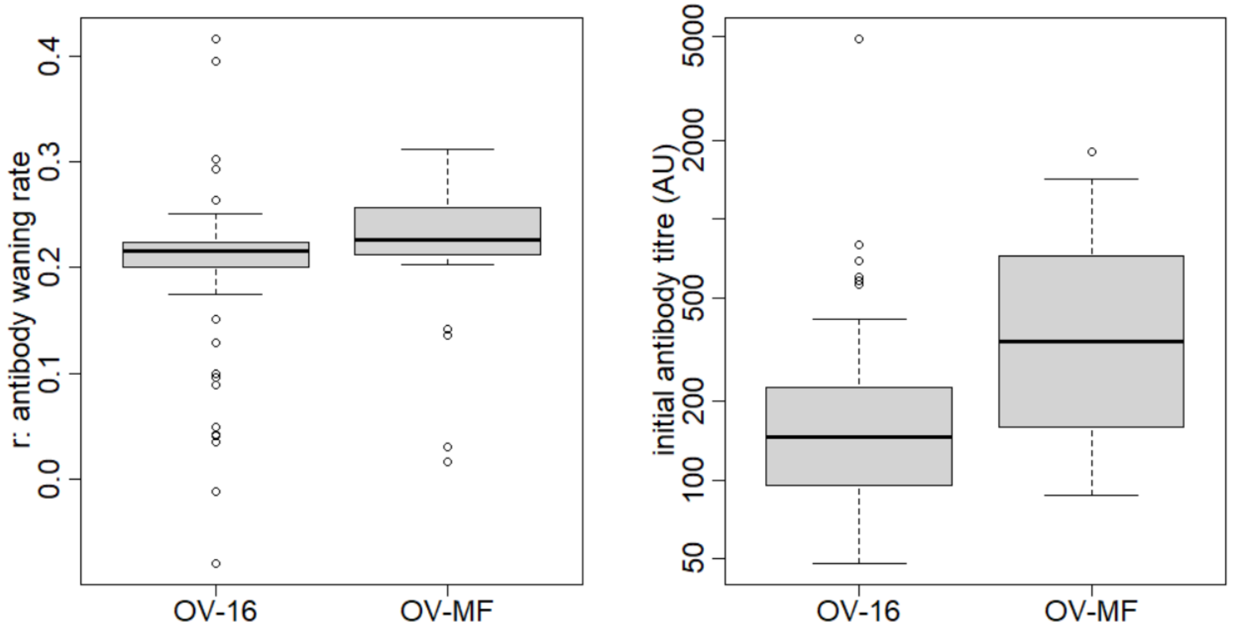

Supplement: Supplemental Materials [file tpmd230473.SD1.pdf]
